# Dry conditions disrupt terrestrial–aquatic linkages in northern catchments

**DOI:** 10.1111/gcb.13361

**Published:** 2016-06-13

**Authors:** Erik J. Szkokan‐Emilson, Brian W. Kielstra, Shelley E. Arnott, Shaun A. Watmough, John M. Gunn, Andrew J. Tanentzap

**Affiliations:** ^1^ Ecosystems and Global Change Group Department of Plant Sciences University of Cambridge Cambridge CB2 3EA UK; ^2^ Department of Biology Queen's University Kingston ON K7L 3J9 Canada; ^3^ School of the Environment Trent University Peterborough ON K9L OG2 Canada; ^4^ Vale Living with Lakes Centre Laurentian University Sudbury ON P3E 2C6 Canada

**Keywords:** climate change, dissolved organic carbon, drought, *Hyalella azteca*, littoral invertebrates, metal toxicity, organic soils, terrestrial organic matter

## Abstract

Aquatic ecosystems depend on terrestrial organic matter (tOM) to regulate many functions, such as food web production and water quality, but an increasing frequency and intensity of drought across northern ecosystems is threatening to disrupt this important connection. Dry conditions reduce tOM export and can also oxidize wetland soils and release stored contaminants into stream flow after rainfall. Here, we test whether these disruptions to terrestrial–aquatic linkages occur during mild summer drought and whether this affects biota across 43 littoral zone sites in 11 lakes. We use copper (Cu) and nickel (Ni) as representative contaminants, and measure abundances of *Hyalella azteca*, a widespread indicator of ecosystem condition and food web production. We found that tOM concentrations were reduced but correlations with organic soils (wetlands and riparian forests) persisted during mild drought and were sufficient to suppress labile Cu concentrations. Wetlands, however, also became a source of labile Ni to littoral zones, which was linked to reduced abundances of the amphipod *H. azteca*, on average by up to 70 times across the range of observed Ni concentrations. This reveals a duality in the functional linkage of organic soils to aquatic ecosystems whereby they can help buffer the effects of hydrologic disconnection between catchments and lakes but at the cost of biogeochemical changes that release stored contaminants. As evidence of the toxicity of trace contaminant concentrations and their global dispersion grows, sustaining links among forests, organic soils and aquatic ecosystems in a changing climate will become increasingly important.

## Introduction

The biogeochemistry of organic soils plays an important role in linking the functioning of aquatic ecosystems to their catchments. The main link between organic soils and aquatic ecosystems is the provision of terrestrial organic matter (tOM). tOM can block UV radiation and strengthen lake thermal stratification (Morris *et al*., 1995; Williamson *et al*., 1996; Tanentzap *et al*., 2007), subsidize food resources for organisms at the base of aquatic food webs (Polis *et al*., 1997; Schindler, 2009; Tanentzap *et al*., 2014) and reduce the bioavailability of contaminants in surface waters (Playle, 1998). A second important link by which organic soils regulate aquatic ecosystems is through the filtration of run‐off water, which keeps contaminants out of receiving surface waters. Organic soils are particularly good at retaining metal contaminants and preventing their release into surface waters, and so wetlands are often intentionally used to improve downstream water quality in contaminated catchments (Crist *et al*., 1996; Brown *et al*., 2000).

Global change is now threatening to disrupt linkages between catchments and aquatic ecosystems, especially in northern ecosystems that hold much of the planet's available freshwater (Schindler & Lee, 2010). Summer droughts are increasing in intensity, duration and frequency (Trenberth, 2011), causing low‐flow conditions that effectively disconnect upland soils from their receiving waters and ultimately reduce concentrations and fluxes of tOM from forests and wetlands (Schiff *et al*., 1998; Clark *et al*., 2005; Sowerby *et al*., 2010). Organic soils that are oxidized when dry generate acids that further suppress tOM by reducing organic carbon solubility, and release metals into pore water and stream flow during rain events (Evans *et al*., 2006; Pennington & Watmough, 2015; Watmough & Orlovskaya, 2015). The release of metal contaminants is exacerbated by low‐flow conditions that provide little dilution. Metals and other contaminants reach toxic levels during pulse exposure (Szkokan‐Emilson *et al*., 2013), and even low naturally occurring concentrations can reduce aquatic food web complexity, resulting in fewer species and shorter food chains (Carlisle & Clements, 2003; Hogsden & Harding, 2012). While the biological consequences of climate‐driven acidification events are well studied (Arnott *et al*., 2001; Durance & Ormerod, 2007; Kowalik *et al*., 2007), the effects of this reduced tOM export coupled with increased contaminant release have largely been overlooked. With aerial transport of metal contaminants over hundreds or thousands of kilometres, this is not a local issue confined to industrial landscapes (Ouellet & Jones, 1983; Bollhöfer & Rosman, 2001; Marx & McGowan, 2011; Csavina *et al*., 2012). Elevated metal concentrations have been observed in streams hundreds of kilometres from emission sources (Adkinson *et al*., 2008). Thus, there is a clear need to better understand the biological consequences of disruptions to the supply of protective tOM and contaminant retention by organic soils under warmer and drier conditions.

Biological communities at the confluence of lakes and their receiving waters are especially sensitive to changes in surrounding catchments. Much of the tOM exported from terrestrial soils is deposited nearshore, and nutrients and contaminants released from catchments are concentrated in littoral areas before dilution into the pelagic zone (Wetzel, 1992). As littoral communities account for approximately 50% of lake productivity and as much as 65% of pelagic fish diet (Vadeboncoeur *et al*., 2002; Vander Zanden & Vadeboncoeur, 2002), changes to terrestrial–aquatic linkages in nearshore sites can severely impact entire food webs.

Here, we test whether mild summer droughts are sufficiently strong to disrupt terrestrial–aquatic linkages and reduce littoral abundances of the amphipod *Hyalella azteca*, a widespread indicator of ecosystem condition and food web production (Barton & Hynes, 1976; France, 1993a). We focus on two of the beneficial terrestrial–aquatic linkages that may be disrupted by drought: (i) the provision of tOM, either as a resource subsidy or suppressor of labile metals, and (ii) the retention of metal contaminants in organic soils. We expect that concentrations of tOM in outflow from organic soils of forests and wetlands will decline during mild drought (linkage 1). However, we expect that its mitigating effect on contaminant lability will be outweighed by increased labile metal concentrations (Cu and/or Ni) in littoral zones from organic soil‐derived releases (linkage 2), thereby reducing littoral *H. azteca* abundances. We now show that abundance of this indicator species within northern lakes is linked to the inputs of tOM and labile metals from organic soils in catchments and how these linkages are disrupted by drought.

## Materials and methods

### Study sites and drought conditions

We studied 11 small (0.16–1.75 km^2^), low nutrient (4–28 μg L^−1^ total P), circumneutral (pH 6.5–7.5) lakes that have been part of *H. azteca* recolonization surveys in the region of Sudbury, Canada, since the early 1990s (Table S1). This area is ideal to test our hypotheses because heterogeneity in smelter‐related metal contamination and recovery from past disturbance has left gradients in metal concentrations, vegetation and soil development among lake catchments (McCall *et al*., 1995; Szkokan‐Emilson *et al*., 2011; Meadows & Watmough, 2012). The Canadian National Agroclimate Information Service (Agriculture and Agri‐Food Canada, 2016) noted below normal precipitation in the Sudbury area during the summers of both 2011 and 2012 (Fig. S1). The National Oceanic and Atmospheric Administration (2015), which synthesizes several drought indices to estimate the degree of drought across North America, defined August of 2011 and 2012 as abnormally dry to moderate drought in the Sudbury area. We therefore refer to this as ‘mild drought’ conditions.

We selected three well‐defined subcatchments in each lake catchment based on the availability of data from past *H*. *azteca* surveys, or in unstudied lakes based on the highest range in forest density and organic soil/wetland cover. Subcatchments were defined as area of land drained by a single discharge stream, and littoral sites were defined as nearshore areas in the lakes downstream of subcatchment discharge streams. We sampled five additional sites below subcatchments in two of the 11 study lakes for a total of 43 littoral sites.

### Sample collection

We collected two water samples from just below the surface at each littoral site at the start and end of the sampling period in August 2012. Samples were filtered with 0.2‐μm Isopore membrane filters. We estimated dissolved organic carbon (DOC) in the samples from known absorbance relationships as a measure of the concentration of tOM. UV absorbance was measured at 320 nm for each sample with an Agilent Cary 60 UV–Vis Spectrophotometer and converted to an absorbance coefficient (*K*_320_) on the basis of the Beer–Lambert law. We then used *K*_320_ to estimate DOC concentration using a regression model derived for 58 lakes in our study region that explained 94% of the variation in DOC (Beauclerc & Gunn, 2001).

We focussed on two representative metal contaminants: Cu, whose speciation is controlled largely by organometal complexation, and Ni, regulated primarily by pH (Watmough & Orlovskaya, 2015). We installed diffuse gradients in thin films (DGTs; Davison & Zhang, 1994) in all sites for 2 weeks to estimate DGT‐labile fractions of these contaminants, which are free ions and those forms that quickly dissociate from organic molecules. These labile fractions have been related to stress and toxicity in aquatic organisms, and DGTs are often used as *in situ* estimates of metal bioavailability (Røyset *et al*., 2005; Martin & Goldblatt, 2007). After collection, DGTs were eluted and acidified with 70% trace grade HNO_3_ following standard procedures (Garmo *et al*., 2003). Labile Cu and Ni concentrations from DGTs (Ni_L_ and Cu_L_) were analysed using an Agilent 810 ICP‐MS. DGT‐accumulated metals were converted to concentrations using formulae and elution efficiencies described by Garmo *et al*. (2003) and diffusion coefficients provided by the manufacturer (DGT Research Ltd., Lancashire, UK). We subtracted the mean concentration of two laboratory blanks from all DGTs.

We measured *H. azteca* abundances at each littoral site with eight modified Hester‐Dendy artificial substrates (hereafter, dendies; North Temperate Lakes LTER, 2005) spaced 1 m apart following transects at 0.25–0.75 m depth up to a maximum of 5 m from shore. Dendies were deployed 1 week prior to DGTs to minimize disturbance and left for 25 days, then sieved to retain all animals >500 μm and preserved in 70% ethanol. *Hyalella azteca* were identified and counted under a dissecting microscope. All samples were collected during August of 2012.

### Disruption to terrestrial–aquatic linkages

We tested whether terrestrial–aquatic linkages were disrupted during drought by comparing labile metal and DOC concentrations in stream water before (spring) and during dry conditions (summer and into the early fall). We expected that the disruption would depend on the prevalence of organic soils in the subcatchment area, so we measured DOC and total metal concentrations in stream water collected every 8 h from representative subcatchments above two of our littoral sites. One of the sites had high wetland (organic soil) influence (21% wetland cover) and one had low wetland influence (1.4% wetland cover), representing the range in wetland cover across the 43 littoral sites (from aerial photograph interpretation: 0% to 21% wetland cover, mean = 5.1%). We then used the biotic ligand model (BLM) to estimate labile (free ion) metal concentrations in stream water from both subcatchments. The BLM is an equilibrium model that estimates metal speciation based on the free ion activity model and the Windermere Humic Aqueous Model (blm version 2.2.3; Paquin *et al*., 2000). Default values of 0.01 μm for stream water sulphide and 10% for humic acid composition were used for all simulations (Di Toro *et al*., 2001). Samples from the two representative sites were collected during a mild summer drought in 2011, but comparable dry conditions and water table declines were observed in 2012 throughout the study area during the *H. azteca* sampling period (Szkokan‐Emilson *et al*., 2014; Fig. S1).

### Modelling consequences of disrupted terrestrial–aquatic linkages in littoral sites

We used the data from our 43 littoral sites to test whether abundances of the indicator species *H. azteca* were positively affected by the provision of tOM (DOC) more strongly than they were negatively affected by contaminants released from organic soils during drought conditions. Our approach used path analysis to describe a hypothesized network of causal connections from subcatchment characteristics to nearshore water chemistry and then to *H. azteca* abundances. Path analysis is analogous to multiple regression and aims to estimate the direction and magnitude of dependencies among a set of connected variables (Legendre & Legendre, 2012).

First, we tested how the provision of tOM by organic soils (linkage 1) varied with wetland area and forest density during the summer drought. The total wetland influence in each subcatchment WL_T_ was estimated with the Tasseled Cap Transformation Wetness Index derived from 11 composite Landsat 5 images taken between 27 March and 5 October 2011 (Crist & Cicone, 1984). This index estimates the average wetness or saturation in pixels across seasons within the subcatchment. Total forest density NDVI_T_ was estimated over the subcatchment by averaging Normalized Difference Vegetation Index (NDVI) values from Landsat 5 images. NDVI estimates vegetation density and biomass based on the absorbance of chlorophyll activity in plants (Pettorelli *et al*., 2005). We also estimated riparian forest density NDVI_R_ by summing NDVI restricted to the area of the subcatchment up to a maximum 100 m from the point of stream discharge into the lake site. Summing accounted for differences in contributing riparian areas across subcatchments and values were not correlated with NDVI_T_ (Pearson *r* = 0.23, *P* = 0.137). We then modelled DOC in each littoral site *i* and lake *j* as a lognormally distributed variable with a mean *α* ^(1)^ across lakes that varied with NDVI_R_, NDVI_T_, and WL_T_, and unobserved error $v_{j}^{\left(1\right)}$ at the lake level:

(1) $${\text{DOC}}_{ij}∼\ln N\left(\mu_{ij},\sigma_{\mathrm{DOC}}\right)$$

$$\mu_{ij}=\alpha^{\left(1\right)}+\beta_{1}{\text{NDVI}}_{Rij}+\beta_{2}{\text{NDVI}}_{Tij}+\beta_{3}{\text{WL}}_{Tij}+v_{j}^{\left(1\right)}.$$

Second, we tested for evidence of a disruption to the retention of contaminants in organic soils (linkage 2) by determining whether labile metals (Ni_L_ and Cu_L_) increased downstream of subcatchments with large wetlands and decreased as more tOM (DOC) was released. Wetlands that were directly connected to littoral sites by an outflow stream were identified from high‐resolution photographs. We used a Compound Topographic Wetness Index overlay to confirm interpretations and identify additional cryptic wetlands (Creed *et al*., 2003) and then distance‐weighted the connected wetland areas by stream length to account for in‐stream run‐off dilution and biogeochemical processes. Metals are more soluble in acidic conditions and so we also accounted for variation among sites in pH. We then modelled Ni_L_ in each littoral site *i* and lake *j* as a lognormally distributed variable with mean *α* ^(2)^ that varied with pH, DOC, the area of wetland influence (WL_C_) and unobserved error $v_{j}^{\left(2\right)}$ at the lake level:

(2) $${\text{Ni}}_{Lij}∼\ln N\left(\mu_{ij},\sigma_{{\mathrm{Ni}}_{L}}\right)$$

$$\mu_{ij}=\alpha^{\left(2\right)}+\beta_{4}{\text{pH}}_{ij}+\beta_{5}{\text{DOC}}_{ij}+\beta_{6}{\text{WL}}_{Cij}+v_{j}^{\left(2\right)}.$$

We fitted the same model as above for Cu_L_, with mean *α* ^(3)^ and unobserved error $v_{j}^{\left(3\right)}$.

Finally, we tested how abundances (Abund_*ijk*_) of *H. azteca* in each dendy *k* at littoral site *i* and lake *j* varied both with tOM (DOC) as a bottom‐up resource subsidy and with labile metals (Cu_L_ and Ni_L_), which may have been suppressed by tOM. We also accounted for known variation in *H. azteca* abundances simply because of the depth of dendy deployment (Lindeman & Momot, 1983). We also included site pH because of its potential effects on abundance (Snucins, 2003). We specifically modelled Abund_*ijk*_ as a Poisson lognormal variable with error term *ɛ*_*ijk*_ accounting for overdispersion (Elston *et al*., 2001), mean *α* ^(4)^ across lakes that varied with Ni_L_, Cu_L_, DOC, Depth, pH and unobserved random variation *v*_*i*_ and $v_{j}^{\left(4\right)}$ at the site and lake level, respectively:

(3) $${\text{Abund}}_{ijk}∼\text{Pois}\left(\lambda_{ijk}\right)$$

$$\lambda_{ijk}=\exp\left(\alpha^{\left(4\right)}+\beta_{10}{\text{Ni}}_{Lij}+\beta_{11}{\text{Cu}}_{Lij}+\beta_{12}{\text{DOC}}_{ij}+\beta_{13}{\text{Depth}}_{ij}+\beta_{14}{\text{pH}}_{ij}+v_{j}^{\left(4\right)}+v_{i}+\varepsilon_{ijk}\right)$$

### Model estimation

Statistical models were fitted in a Bayesian framework with Markov chain Monte Carlo (MCMC) sampling using rstan v.2.9.0 (Stan Development Team, 2016) in r v.3.2.2 (R Core Team, 2015). Four MCMC chains of 3500 iterations were simulated for each model, with a burn‐in of 1000 runs. We assigned uninformative priors of distribution *N*(0, 10) for coefficients *α* and *β* and *U*(0, 10) for *σ*'s. Random variation at the site and lake level was also drawn from zero‐mean normal distributions but with separately estimated SDs. Model convergence and mixing of MCMC chains was verified visually with trace plots and through two diagnostic measures. Firstly, we calculated the potential scale reduction factor $\hat{R}$, which predicts the extent to which a parameter's credible intervals (CIs) will be reduced if models are run for an infinite number of simulations (Gelman & Hill, 2007). Secondly, we calculated the effective sample sizes *n*_eff_, as a measure of independence among simulations (Gelman & Hill, 2007). For all models, $\hat{R}$ values were <1.1 and *n*_eff,_ values were over 700 indicating MCMC mixing and model convergence (Gelman & Hill, 2007).

To infer effects, we calculated posterior means and 95% CIs for each parameter by drawing a subset of 1000 simulations from the four chains. Tested linkages in our path analysis were considered supported if 95% CIs around estimated effect sizes (*β*'s) excluded zero. As our interest was in within‐lake processes, we summarized model fit with a *R* ^2^ that calculated the proportional change in observation‐level (or within‐lake) residual variance between full and null (intercept) models (Nakagawa & Schielzeth, 2013), but conditional *R* ^2^'s were also calculated. Residual variance for the Poisson lognormal model was calculated as the sum of distribution‐specific variance and overdispersion variance (Nakagawa & Schielzeth, 2010).

## Results

### Drought disrupts terrestrial–aquatic linkages

**Figure 1:**
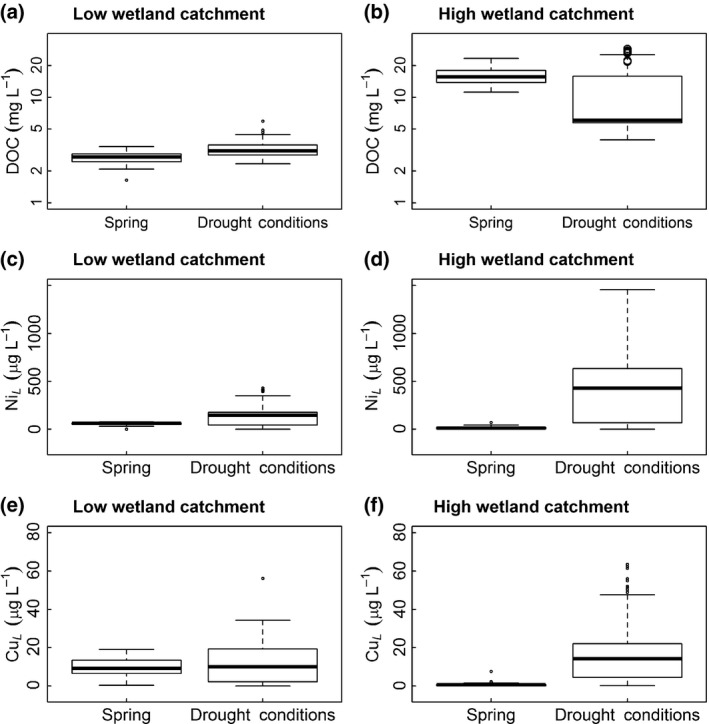
Drought reduces dissolved organic carbon (DOC) export but elevates metal release in catchments with high wetland influence. DOC concentrations increase minimally from the low wetland (1% wetland cover) catchment (a) but decrease from the high wetland (21% wetland cover) catchment (b). Labile metal concentrations (Ni_L_, Cu_L_) show minimal change from the low wetland catchment (c, e) vs. large increases from the high wetland catchment (d, f). Open circles in panel (b) indicate an increase in DOC observed during the onset of drought.

We found that the provision of tOM from organic soils (terrestrial–aquatic linkage 1) was disrupted during mild summer drought within the high coverage but not the low coverage wetland subcatchment. Following a brief increase at the onset of the drought, DOC concentrations decreased significantly from the subcatchment with high wetland influence [before: mean (SD)* *= 16.1 (3.1) mg L^−1^, after: mean (SD) = 9.5 (6.1) mg L^−1^, *t*‐test: *t*_113_ = −7.94, *P* < 0.001], suggesting that the oxidation/acidification of organic soils reduced organic carbon solubility. This is in contrast to a slight increase at the subcatchment with low wetland influence, as would be expected where oxidation of organic soils is not a dominant process, because of increased decomposition (and DOC formation) in warm summer conditions [mean (SD): before = 2.66 (0.45) mg L^−1^, after = 3.27 (0.64) mg L^−1^, *t*_95_ **= 3.64, *P* < 0.001]. Despite the decrease in DOC at the high wetland site, average concentrations remained nearly four times higher than the site with low wetland influence, indicating some maintenance of tOM provision despite the disruption of this linkage (Fig. 1a, b).

We also found that the retention of contaminants in organic soils (terrestrial–aquatic linkage 2) was disrupted during mild summer drought, thereby releasing labile Ni and Cu. This response was larger in the high wetland subcatchment, where average labile Ni concentrations increased by 36 times [mean (SD): before = 12.9 (13.4) μg L^−1^, after = 470 (437) μg L^−1^, *t*_82_ = 9.53, *P *< 0.001], compared to 2.7 times from the low wetland subcatchment [mean (SD): before* *= 55.2 (18.5) μg L^−1^, after = 147 (124) μg L^−1^, *t*_95_ = 2.95, *P* = 0.004] (Fig. 1c, d). Similarly, labile Cu concentrations increased 26 times in outflow from the high wetland subcatchment [mean (SD): before* *= 0.75 (1.11) μg L^−1^, after = 19.6 (17.1) μg L^−1^, *t*_74_ = 9.43, *P *< 0.001], but did not change beneath the low wetland site [mean (SD): before* *= 9.85 (5.01) μg L^−1^, after = 11.6 (10.7) μg L^−1^, *t*_95_ = 0.65, *P* = 0.515] (Fig. 1e, f).

### Consequences of disrupted terrestrial–aquatic linkages in littoral sites

**Figure 2:**
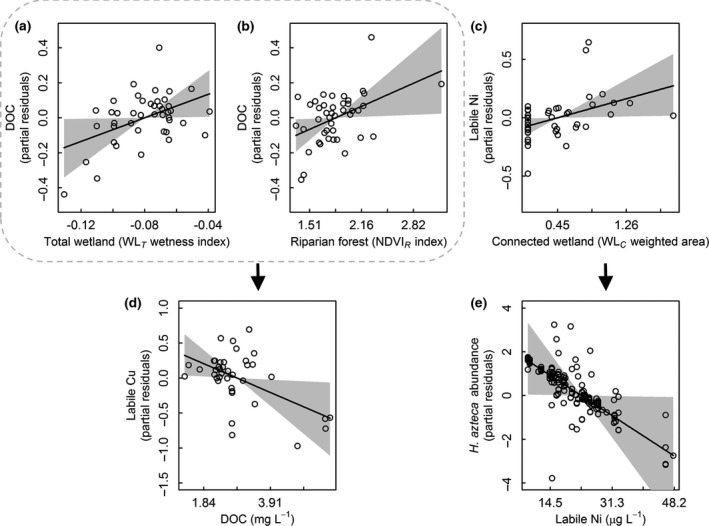
Disrupted terrestrial–aquatic linkages during mild drought conditions. Terrestrial organic matter (tOM) concentration dissolved organic carbon (DOC) increases with total wetland (a) and riparian forest density (b) and in turn suppresses labile Cu concentration (d). Labile Ni increases with connected wetland area (c), which in turn suppresses *Hyalella azteca* abundances (e). Lines indicate mean model fit ±95% CI (grey polygons). Points are (a–d) site‐ or (e) dendy‐level partial residuals. Model statistics are reported in Table 1.

Patterns of DOC in downstream littoral sites further supported our prediction that drought would disrupt terrestrial–aquatic linkage 1. DOC concentrations were relatively low during the drought, ranging from 1.24 to 6.22 mg L^−1^ across the 43 littoral sites (Table 1), and increased with subcatchment wetland area (Table 2, Fig. 2a), consistent with our observations in stream outflows (Fig. 1a, b). We also found that DOC concentration increased in littoral sites with riparian forest area (Table 2, Fig. 2b).

**Table 1:** Mean (SD) of labile metal (Cu_L_, Ni_L_) and DOC concentrations in all littoral sites, and compared among sites without *Hyalella azteca* (average dendy abundance below 1 animal), those with some present (from 1 to 31 animals) and those with high abundance (at least 32 animals, i.e. above the 85th percentile of abundance)

	Overall	Range	*H. azteca* abundance group		
			Absent	Present	Abundant
Cu_L_ (μg L^−1^)	1.32 (0.52)	0.37–3.06	1.19 (0.44)	1.58 (0.64)	1.25 (0.32)
Ni_L_ (μg L^−1^)	22.8 (8.32)	8.38–47.9	26.0 (8.78)	20.7 (4.08)	16.5 (8.79)^a^
DOC (mg L^−1^)	3.12 (1.31)	1.24–6.22	3.02 (1.21)	3.03 (1.25)	3.62 (1.77)
pH	6.27 (0.81)	3.86–7.94	6.06 (0.52)	6.48 (1.22)	6.53 (0.14)
Sites (*N*)	43	43	22	14	7

a Significant difference from sites without *H. azteca t*_95_ = −2.85, *P* = 0.007.

**Table 2:** Estimated effects for terrestrial–aquatic linkage models

Response	Mean parameter estimates (95% CI)		Within‐lake (conditional) *R* ^2^
	Significant effects	Nonsignificant effects	
DOC^1^	**Riparian forest density 0.066 (0.006–0.126)**	Total forest density −0.002 (−0.083 to 0.078)	0.29 (0.87)
	**Total wetland 0.067 (0.003–0.134)**		
Labile Ni^2^	**Connected wetland 0.082 (0.006–0.163)**	DOC −0.098 (−0.298 to 0.111)	0.31 (0.78)
		pH −0.044 (−0.160 to 0.067)	
Labile Cu^3^	**DOC −0.205 (−0.397 to −0.023)**	pH 0.054 (−0.114 to 0.199)	0.25 (0.43)
		Connected wetland −0.003 (−0.144 to 0.130)	
Abundance^4^	**Labile Ni −0.914 (−1.879 to −0.041)**	Labile Cu 0.629 (−0.086 to 1.371)	0.94 (0.91)
	**Depth −1.227 (−1.915 to −0.575)**	DOC 0.016 (−1.064 to 1.087)	
		pH −0.149 (−0.908 to 0.651)	

Model fit is shown as a within‐lake *R* ^2^ calculated at the observation level along with conditional *R* ^2^ in brackets (see text for details of calculation). Significant effects (not overlapping zero) are bolded.

See supplementary table ^1^S2a, ^2^S2b, ^3^S2c and ^4^S2d for full model details.

**Figure 3:**
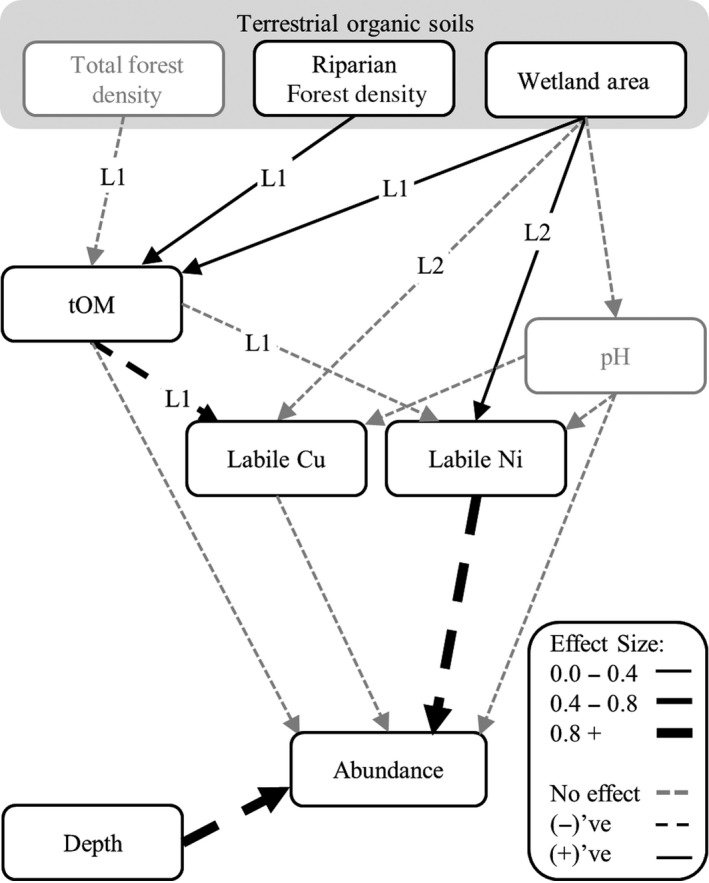
Path analysis of linkages between terrestrial organic soils and aquatic biota (*Hyalella azteca* abundances), as disrupted by drought. Model effect sizes are shown for the two linkages: (L1) the provision of terrestrial organic matter (tOM; measured as DOC) as a regulator of contaminant lability, and (L2) the supply of labile metal contaminants from organic soils. Wetland area is either total wetland influence (in L1 models) or connected wetland area (in L2 models). Parameters with no significant relationships are greyed. Model statistics and parameters are reported in Table 1.

Labile metals in the littoral sites also supported our prediction of a disruption to linkage 2 during drought that released contaminants from soils (Fig. 3). Labile Ni ranged from 8.38 to 47.9 μg L^−1^ across the littoral sites (Table 1) and increased with connected wetland area (Table 2, Fig. 2c). This was consistent with the BLM results predicting some labile Ni release during drought even from the subcatchment with low wetland influence, and higher release from the subcatchment with high wetland influence (Fig. 1c, d). In contrast to Ni, labile Cu concentrations were lower, ranging from 0.37 to 3.06 μg L^−1^ across the littoral sites (Table 1), and did not increase with weighted wetland area (Table 2). This was again consistent with the BLM results that showed no detectable increase in release from the subcatchment with the smaller wetland (Fig. 1e, f). Partly, tOM continued to suppress Cu, as labile concentrations were negatively associated with DOC (Table 2, Fig. 2d). There was no detectable effect of pH on labile Ni or Cu concentrations in the littoral sites (Table 2).

Given disruption to both terrestrial–aquatic linkages during the drought, we found that *H. azteca* abundances were substantially reduced, as there was a negative association between abundance and labile Ni that was mediated through these linkages (Fig. 3). For example, a 2 SD increase in wetland influence above average conditions reduced *H. azteca* abundances by 49% through the soil Ni linkage (disrupted linkage 2; Fig. 3). Despite evidence of reductions in DOC from large wetlands during drought (disrupted linkage 1; Fig. 1b), the provision of tOM from wetland and riparian forest soils remained sufficient to reduce labile fractions of Cu, which has a higher affinity for DOC, to levels that did not influence *H. azteca* abundances (Table 2, Fig. 2a, b, d). The lack of a direct effect of DOC on *H. azteca* also suggested that tOM was not acting as a bottom‐up resource subsidy during the summer drought (Table 2). Aside from these linkages, we also found fewer animals in deeper waters (Table 2).

## Discussion

Here, we have found strong evidence, and the first to our knowledge, that important linkages between lake biota and terrestrial organic soils can be disrupted even during mild summer droughts. These findings have the potential to transform the way we view terrestrial–aquatic linkages by revealing a striking duality in the function of organic soils during dry conditions. We specifically found that organic soils can buffer the effects of hydrologic disconnection between catchments and lakes, but at the cost of biogeochemical changes that release stored metal contaminants that harm biota in receiving waters. This extends previous work, which has shown drought impairs aquatic invertebrate colonization by inducing acidification events (Arnott *et al*., 2001; Durance & Ormerod, 2007; Kowalik *et al*., 2007). In littoral zones, the mixing of stream outflow and lake water with higher buffering capacity can regulate these pH impacts; however, released metal contaminants may also create lingering effects that extend beyond dry conditions because they can precipitate and accumulate with deposited littoral sediments (McKnight & Bencala, 1990; Roulier *et al*., 2008). Our results therefore suggest that more frequent and intense dry conditions brought on by climate change will disrupt terrestrial–aquatic linkages and potentially damage lake food webs.

### Disruption to terrestrial–aquatic linkages

We found evidence that the provision of tOM from organic soils (terrestrial–aquatic linkage 1) was disrupted by drought. Concentrations of DOC in streams draining wetlands were reduced during drought, which is counter to what is expected in warm summer conditions when decomposition and DOC production from organic soils should be highest (Freeman *et al*., 2001; Evans *et al*., 2006), and dilution of DOC by overland flows should be lowest (Eimers *et al*., 2008; Sowerby *et al*., 2010). However, similar reductions in DOC concentration in response to drought have been observed elsewhere (Clark *et al*., 2005, 2011) and have been attributed to either decreased solubility brought on by increased acidity and ionic strength or perhaps enhanced oxidative decomposition to CO_2_ (Pastor *et al*., 2003; Clark *et al*., 2005, 2011). Regardless of the cause of the decline, the results supported our hypothesis that there is some continued provision of tOM through the drought, as DOC concentrations in littoral sites remained correlated with wetland and riparian forest areas and lent to the suppression of labile Cu concentrations.

We also found evidence that the retention of contaminants by organic soils (terrestrial–aquatic linkage 2) was disrupted by drought causing wetlands to become a source of metals to littoral sites. This reveals a duality of organic soil function during dry conditions, whereby there is a benefit of tOM provision but at the cost of an added contaminant release. Previous studies have demonstrated similar drying and oxidation‐related increases in metal concentrations from organic wetland soils (Tipping *et al*., 2003; Juckers & Watmough, 2014), with concentrations exceeding water quality guidelines by orders of magnitude in catchments where large stores of metal contaminants have accumulated (Szkokan‐Emilson *et al*., 2013). Although our sites are in an area with relatively high smelter‐related Cu and Ni concentrations, similar metal releases have been observed at sites almost 300 km from contaminant sources (Adkinson *et al*., 2008). We show here that the geochemical effects of these dry conditions extend out into littoral zones with the potential to damage aquatic biota in receiving waters.

Terrestrial organic matter can provide a direct food source for some consumers (Cole *et al*., 2006; Bartels *et al*., 2012) or subsidize microbial communities that then feed higher trophic levels (Jansson *et al*., 2007; Tanentzap *et al*., 2014), but we found no effect of tOM on abundances on *H. azteca*. Although there was evidence that the supply of tOM was reduced, concentrations were still within a range observed to promote heterotrophic bacterial biomass (Tanentzap *et al*., 2014). As we hypothesized, the toxicity resulting from disrupted linkages may have outweighed the potential tOM subsidy effect because both tOM and labile metal concentrations were concurrently highest in littoral sites downstream of large wetlands. However, increased inputs of highly recalcitrant tOM can also reduce productivity in lakes by altering physical (e.g. light and temperature) and chemical (e.g. nutrient and oxygen) conditions (Stasko *et al*., 2012; Kelly *et al*., 2014; Karlsson *et al*., 2015). DOC concentrations in our study were within a range observed to shade out and reduce primary productivity (Thrane *et al*., 2014), so it is possible that tOM elicited both positive and negative effects and we could only detect the net outcome in our models.

### Potential effects on aquatic food webs

Our results suggest that aquatic food webs may suffer from disruption to terrestrial–aquatic linkages during summer droughts. We found that the abundance of *H. azteca* decreased with labile Ni concentrations in littoral sites, which were within ranges that are chronically toxic. Schroeder *et al*. (2010) found 28 day toxicity of free ion Ni (LC50s) to *H. azteca* to average 44.6 μg L^−1^ and as low as 17.6 μg L^−1^. Our sites had labile concentrations in this range, from 12.5 to 46.2 μg L^−1^, with conditions persisting longer than 28 days, even though the source of the pollutants has been greatly reduced for over 30 years (Szkokan‐Emilson *et al*., 2013). These reductions in abundance are notable because *H. azteca* can account for as much as 65% of fish diet (Jansen & Mackay, 1992; Vander Zanden & Vadeboncoeur, 2002). *Hyalella azteca* is also one of the most ubiquitous benthic invertebrates in freshwater systems (Lindeman & Momot, 1983; France, 1993b), making any reduction in their abundances likely to influence entire food webs.

Although we chose Ni and Cu as representative contaminants, the toxic effect of these disrupted linkages would actually be the product of many interactive and correlated contaminants. For example, Watmough & Orlovskaya (2015) found Co, Mn and Zn to be released along with Ni from peatland soils in response to drying, and all of these metals are chronically toxic to *H. azteca* and other aquatic organisms (Borgmann *et al*., 2005; Norwood *et al*., 2007). Although the lability of Cu remained low because of its high affinity with DOC and organic soils (Santore *et al*., 2001; Novak *et al*., 2011), there are several other metals that are regulated by organic matter to varying degrees and some are toxic at low concentrations [e.g. Pb and Cd; Borgmann *et al*. (2005)]. Furthermore, other keystone invertebrates such as *Hexagenia* spp. and *Ceratodaphnia pulex* are equally or more sensitive to certain metals than *H. azteca* (Milani *et al*., 2003; Keithly *et al*., 2004), so the potential for impacts to aquatic communities is great. As evidence of the toxicity of even trace concentrations (Carlisle & Clements, 2003; Hogsden & Harding, 2012) and the extent of global dispersion of metals grows (Steinnes & Friedland, 2006; Marx & McGowan, 2011; Csavina *et al*., 2012), it is increasingly important that we better understand the biogeochemical links among forests, organic soils and aquatic ecosystems in a changing climate.

## Supporting information


**Table S1.** Latitude and longitude of the 11 study lakes.
**Table S2.** Estimated effects for terrestrial–aquatic linkage models.
**Figure S1.** Map of Ontario precipitation relative to climate normals in 2011 and 2012.
