# Supplementary material for: Dry conditions disrupt terrestrial–aquatic linkages in northern catchments
Source: Glob Chang Biol. 2016 Jun 13;23(1):117–26. doi: 10.1111/gcb.13361 (PMC6849552; doi:10.1111/gcb.13361)
Supplement: Supplementary file 1 — Table S1. Latitude and longitude of the 11 study lakes. Table S2. Estimated effects for terrestrial–aquatic linkage models. Figure S1. Map of Ontario precipitation relative to climate normals in 2011 and 2012. [file GCB-23-117-s001.pdf]

Table S1: Latitude and longitude of the 11 study lakes.

Lake	Latitude	Longitude
Broder23	-80.95676	46.39693
Clearwater	-81.05049	46.37041
Daisy	-80.88600	46.45168
Dill	-80.90170	46.38967
Forest	-80.99619	46.39233
Hannah	-81.03831	46.44332
Laurentian	-80.95582	46.44953
Little Raft	-80.96871	46.40023
Lohi	-81.04332	46.38755
Middle	-81.02488	46.43903
Raft	-80.94536	46.40967

Table S2: Estimated effects for terrestrial-aquatic linkage models. Predictors with confidence interval of beta estimates which exclude zero are bolded.

(a) tOM (ln(DOC)) model

**Parameter**	**Mean estimate (95% CI)**
Intercept ($\alpha^{(1)}$)	1.144 (0.897 - 1.391)
**NDVI$_R$ (β_1)**	**0.066 (0.006 - 0.126)**
NDVI$_T$ (β_2)	−0.002 (−0.083 - 0.078)
**WL$_T$(β_3)**	**0.067 (0.003 - 0.134)**
σ among lakes $v_j^{(1)}$	0.382 (0.230 - 0.673)

(b) Labile Nickel (ln(Ni$_L$)) model

**Parameter**	**Mean estimate (95% CI)**
Intercept ($\alpha^{(2)}$)	3.023 (2.783 – 3.258)
pH (β_4)	–0.044 (–0.160 - 0.067)
DOC (β_5)	–0.098 (–0.298 - 0.111)
***WL$_C^{(1/4)}$* (β_6)**	**0.082 (0.006 - 0.163)**
σ among lakes $v_j^{(2)}$	0.365 (0.195 - 0.665)

(c) Labile Copper (ln(Cu$_L$)) model

**Parameter**	**Mean estimate (95% CI)**
Intercept ($\alpha^{(3)}$)	0.200 (0.001 - 0.430)
pH (β_7)	0.054 (–0.114 - 0.199)
**DOC (β_8)**	**−0.205 (−0.397 - −0.023)**
WL$_C^{(1/4)}$ (β_9)	–0.003 (–0.144 - 0.130)
σ among lakes $v_j^{(3)}$	0.223 (0.035 - 0.526)

(d) Abundance model

**Parameter**	**Mean estimate (95% CI)**
Intercept ($\alpha^{(4)}$)	0.032 (−1.755 - 1.754)
**Ni$_L$ (B_{10})**	**−0.914 (−1.879 - −0.041)**
Cu$_L$ (β_{11})	0.629 (−0.086 - 1.371)
ln(DOC) (β_{12})	0.016 (−1.064 - 1.087)
**Depth (β_{13})**	**−1.227 (−1.915 - −0.575)**
pH (β_{14})	−0.149 (−0.908 - 0.651)
σ among lakes $v_j^{(4)}$	2.569 (1.368 - 4.817)
σ among sites v_i	1.379 (0.888 - 2.026)
σ overdispersion $\mathcal{E}_{ijk}$	0.620 (0.491 - 0.793)

(e) pH model (alternative linkage model tested)

**Parameter**	**Mean estimate (95% CI)**
Intercept $(\alpha^{(4)})$	6.269 (5.671 – 6.824)
$WL_{C}^{(1/4)}$ (β_{15})	−0.021 (−0.198 - 0.173)
σ among lakes $v_{j}^{(5)}$	0.858 (0.458 – 1.536)

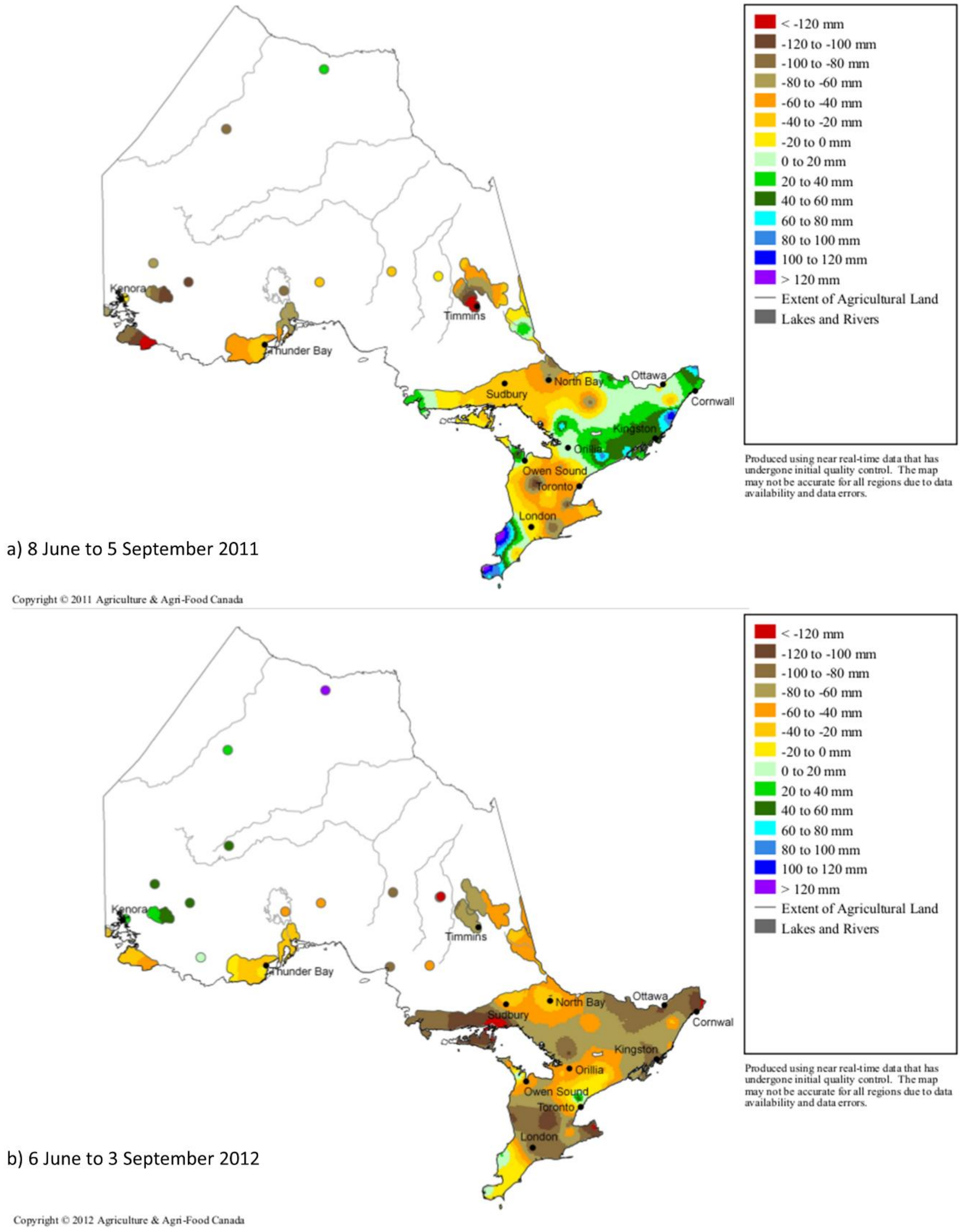

Figure S1: Map of Ontario precipitation relative to climate normals, showing below normal precipitation in the Sudbury area during the summers of 2011 (a) and 2012 (b). Reproduced from National Agroclimate Information Service maps (Agriculture and Agri-Food Canada 2016).
